# Incidence of Discordant Pleural Fluid Exudates and Diagnostic Patterns: A Retrospective Cohort Study

**DOI:** 10.1016/j.chest.2025.05.048

**Published:** 2025-06-28

**Authors:** Dinesh N. Addala, Rachel Mercer, Anand Sundaralingam, Beenish Iqbal, Alguili Elsheikh, Eihab O. Bedawi, John Wrightson, Robert J. Hallifax, Najib M. Rahman

**Affiliations:** a Oxford Pleural Unit, Oxford University Hospitals, Oxford, United Kingdom; b Oxford Respiratory Trials Unit, Nuffield Department of Medicine, University of Oxford, Oxford, United Kingdom; c Oxford NIHR Biomedical Research Centre, Oxford University, Oxford, United Kingdom; d Portsmouth Hospitals University NHS Trust, Portsmouth, United Kingdom; e Department of Respiratory Medicine, Brearley Wing, Northern General Hospital, Sheffield Teaching Hospitals NHS Foundation Trust, Sheffield, United Kingdom

**Keywords:** diagnostics, discordant exudates, pleural, pleural fluid biochemistry

## Abstract

**Background:**

Light’s criteria use pleural fluid protein and lactate dehydrogenase (LDH) to differentiate pleural effusions as exudative or transudative. In a subset of exudative pleural effusions, discordance occurs between LDH and protein (ie, protein high, LDH low, or vice versa).

**Research Question:**

What incidence and diagnostic profile are associated with discordant pleural fluid biochemistry?

**Study Design and Methods:**

We conducted a retrospective analysis of 995 pleural fluid samples between 2015 and 2017 from a UK tertiary center. Exudates were subdivided into concordant or discordant, with low protein defined as < 30 g/L and low LDH as < 170 IU/L. Demographic characteristics and diagnostic patterns were assessed in both groups. A χ^2^ test and ORs (± 95% CI) were calculated for each diagnosis between discordant and concordant pleural effusions, and adjusted ORs were calculated by using multivariable logistic regression.

**Results:**

In 715 exudative pleural fluid samples, 229 (32%) were discordant. Eighty-five (37%) of these displayed low protein, with high LDH, and 144 (63%) displayed low LDH with high protein. The median age was higher in the discordant group than in the concordant group (75 years vs 70 years; *P* = .01). The proportion of patients with the following diagnoses were significantly higher in the discordant group compared with the concordant group: fluid overload (10% [24 of 229] discordant vs 2% [10 of 486] concordant; *P* < .0001), benign asbestos-related pleural effusion (14% [33 of 229] vs 9% [44 of 486]; *P* = .031), and ICU-associated effusion (9% [20 of 229] vs 3% [15 of 486]; *P* = .001). The following were less frequent in the discordant group: pleural infection (6% [14 of 229] vs 16% [79 of 486]; *P* < .0001) and malignant pleural effusion (34% [77 of 229] vs 42% [206 of 486]; *P* = .025). These patterns were maintained when adjusting for age and sex.

**Interpretation:**

Our results indicate that discordant pleural effusions are common and represent a biologically distinct entity with different diagnostic patterns compared with concordant effusions. Clinicians should assess for discordance early and tailor investigations accordingly.

## Graphical Abstract

**Figure:**
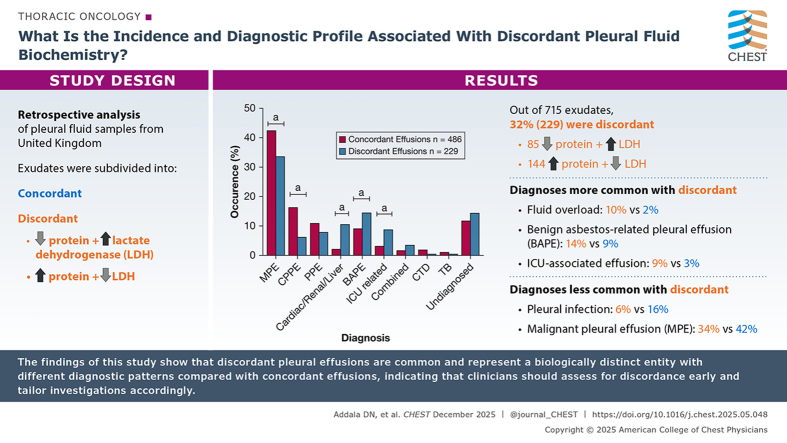

FOR EDITORIAL COMMENT, SEE PAGE 1293

**Take-Home Points**

**Study Question:** What is the incidence of discordant pleural fluid exudates (those with high protein but low lactate dehydrogenase [LDH] or vice versa), and do these exhibit differing diagnostic patterns compared with concordant pleural exudates (high protein, high LDH)?

**Results:** More than 30% of analyzed exudative pleural fluid samples were discordant. Discordance was associated with significantly higher rates of fluid overload, benign asbestos-related pleural effusion, and ICU-associated pleural effusion; discordance was significantly less common in pleural infection and malignant pleural effusion.

**Interpretation:** Discordant pleural exudates represent a common clinical problem and follow different diagnostic paradigms vs concordant exudates. As such, clinicians should tailor their investigations and diagnostic strategies accordingly in the presence of discordant pleural fluid biochemistry.

Pleural effusions represent an increasingly common clinical presentation, with an incidence of approximately 200,000 new cases in the United Kingdom per year,^1^ with > 50 known causes.^2^ The differential diagnosis in this patient cohort is often wide, with early sampling of the pleural fluid and biochemical analysis forming the initial steps in the diagnostic pathway.^3^^,^^4^ Light’s criteria have been used for > 50 years to distinguish pleural fluid as exudative or transudative.^5^ These well-known criteria classify the following as exudate: (1) pleural fluid protein: serum protein ratio > 0.5; (2) pleural fluid lactate dehydrogenase (LDH): serum LDH > 0.6; or (3) pleural fluid LDH is two-thirds the normal serum LDH, per reference range for the laboratory. Exudative effusions occur typically due to local factors such as inflammation of the pleura, most commonly in conditions such as pneumonia/infection, malignancy, and TB. Transudative effusions occur due to imbalances between oncotic and hydrostatic forces, resulting in ultrafiltration across a membrane in conditions such as cardiac failure, liver cirrhosis, and renal impairment.^6^

Previous studies have shown that Light’s criteria misclassify approximately 25% of transudates as exudates,^7^ increasing the potential for delays in both accurate diagnosis and treatment. Furthermore, a subset of exudative pleural effusions (according to Light’s criteria) display discordance between pleural fluid LDH and protein,^8^ wherein the pleural fluid LDH is high but the protein is low or vice versa. The clinical diagnoses and pathophysiology underlying discordant exudates are not well known, and this represents an area of uncertainty in pleural effusion diagnostics. With increasing access to pleural specialist services leading to earlier sampling of pleural effusions in clinical practice, improving clinician understanding of the factors and diagnoses leading to biochemically discordant pleural effusions is essential.

This aim of the current study was to establish the incidence of pleural fluid exudates that displayed discordance between LDH and protein. Further analysis was conducted to establish the demographic characteristics and underlying clinical diagnoses leading to discordance. We hypothesized that distinct diagnostic entities result in discordant biochemistry, and early analysis beyond Light’s criteria to identify discordant exudates could lead to more targeted investigations and facilitate earlier diagnosis of the underlying condition in undiagnosed pleural effusions.

## Study Design and Methods

The use of the data set for this study was approved by the UK Health Research Authority (IRAS ID 275779), and the need for informed consent on a per-patient basis was waived.

### Clinical Data Collection

Retrospective analysis of all pleural fluid results from Oxford University Hospitals (a UK tertiary center) between January 1, 2015, and December 31, 2017, was undertaken. Identification of relevant cases was achieved through a central reporting database for pleural procedures, done by either the respiratory department or radiology department. To avoid duplication, the pleural fluid result used was the first sample sent for an individual patient for diagnosis. Subsequent samples were not included as new cases, but information from repeat aspirates was used to determine final diagnosis.

Samples determined as transudative based on low pleural fluid protein, low LDH, and underlying diagnosis were excluded from final analysis. Samples with incomplete pleural fluid biochemistry were excluded from the complete case analysis but were accounted for, as discussed in the Sensitivity Analysis section. Exudative samples were then divided into concordant and discordant exudates and further subdivided into protein-high discordant exudates and LDH-high discordant exudates.

Data to determine final diagnoses, underlying cause of pleural effusion, and demographic data including sex and age were collected via the hospital’s electronic patient record. This record included procedure reports, radiology, clinic letters, multidisciplinary meeting outcomes, and biochemical and cytology results for pleural fluid in addition to any biopsies. All final diagnoses were determined on the basis of pleural multidisciplinary team discussion. The data were analyzed by 2 separate pleural physicians, and any disagreements in final diagnosis were further checked by a third physician. Data were included until patient death or transfer of care from the trust. Patients were excluded from specific analyses in which information regarding final diagnosis or pleural fluid characteristics was unavailable.

### Definitions

For the purposes of identifying discordant effusions, low pleural fluid protein was defined as < 30 g/dL and low LDH was defined as < 170 IU/L, proportionate to local laboratory normal ranges and consistent with methodology from previous studies.^9^^,^^10^ Exact values differed from prior studies due to variation in normal laboratory ranges. Protein-high discordant exudates were defined as those with protein > 30 g/dL and LDH < 170 IU/L (ie, protein high but LDH low). LDH-high discordant exudates displayed LDH > 170 g/dL and protein < 30 g/dL (ie, LDH high but protein low).

### Statistical Analysis

Descriptive statistics were calculated as frequency (percentages) for categorical variables, and medians and interquartile ranges (IQRs) were used for continuous variables. The distribution of continuous variables was assessed by using the Shapiro-Wilk test. All continuous variables showed non-normal distribution (Shapiro-Wilk, *P* < .05), except for pleural fluid protein in discordant effusions (*P* = .60). However, for consistency, nonparametric tests were applied for all comparisons.

The prevalence of each diagnosis was recorded within each pleural fluid pattern (discordant/concordant). Intergroup comparisons of categorical data were performed by using the χ^2^ test, and ORs were calculated for each diagnosis between discordant and concordant pleural effusions.

ORs were calculated as the odds of a specific diagnosis with discordant pleural effusion divided by the odds of the given diagnosis with concordant pleural effusion. As such, ORs were reported as the OR of a diagnosis occurring in the presence of discordant pleural fluid biochemistry (vs concordant pleural fluid biochemistry). ORs are presented as the OR ± 95% CI.

To adjust for demographic differences between groups, a multivariable logistic regression analysis was undertaken to assess OR ± 95% CI, adjusted for sex and age.

All statistical analysis was performed by using GraphPad Prism version 9 (GraphPad Software) and STATA for Mac version 18 (StataCorp).

### Missing Data

Missing pleural fluid biochemistry result was observed in 89 cases. A sensitivity analysis was conducted on cases with missing pleural fluid protein or LDH values, which could not be determined as concordant or discordant. Both conservative (all missing as concordant) and liberal (all missing as discordant) values were generated and analyzed by using the multivariable logistic regression model.

## Results

### Patient Selection

**Figure 1:**
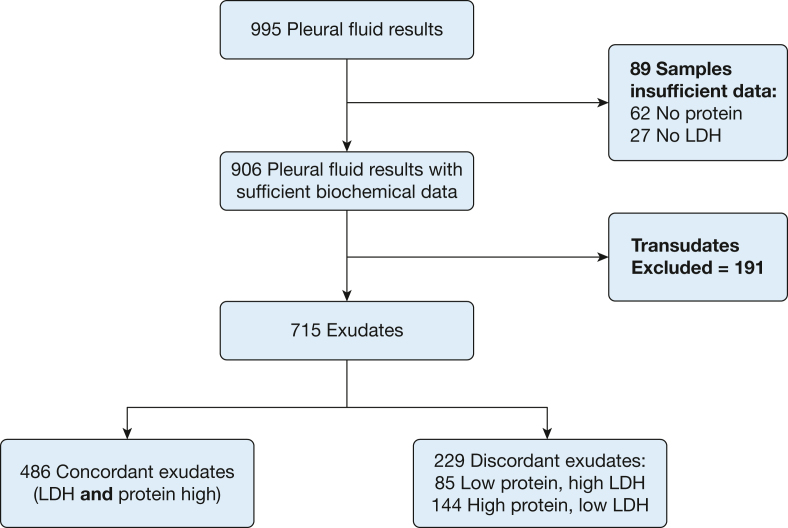
Flowchart illustrating the total numbers of pleural fluid samples and cases analyzed. A total of 715 cases were included in the complete case analysis. LDH = lactate dehydrogenase.

For the duration of the study period, a total of 995 pleural samples qualified for analysis. Sixty-two samples had no data for protein, and 27 had no LDH results; these samples were excluded from the complete case analysis but addressed further in the sensitivity analysis (e-Table 1). A total of 191 samples were transudative and are not described further. Therefore, a total of 715 samples were exudative based on Light’s criteria and were included in the final complete case analysis. Figure 1 illustrates the flow of included samples.

### Demographic Characteristics

Both age and sex were significantly associated with discordant pleural effusions. The median age was higher in the discordant group (75 years vs 70 years; *U* test, *P* = .0002). In logistic regression, older age (OR, 1.02 per year; 95% CI, 1.01-1.03; *P* = .001) and female sex (OR, 1.68; 95% CI, 1.23-2.31; *P* = .001) were independently associated with discordant effusions.

### Concordant Effusions

Of the 715 effusions, 486 (68%) displayed concordance between pleural fluid protein and LDH (both high). The median protein value in these samples was 43 g/L (IQR, 37-47 g/L), and median LDH was 494 IU/L (IQR, 283-908 IU/L). The most common diagnoses in the concordant group were malignant pleural effusion (MPE), parapneumonic effusion, and benign asbestos-related pleural effusion (BAPE). The most common malignancies were lung, mesothelioma, and breast.

### Discordant Effusions

A total of 229 (32%) of 715 patients had biochemistry illustrating discordance between pleural fluid protein and LDH. Of these 229 patients, 144 (63%) were protein-high discordant, and 85 (37%) were LDH-high discordant. The median protein in all discordant effusions was significantly lower than in the concordant group (35 g/L [IQR, 27-42 g/L]; *P* < .0001), and median LDH was significantly lower in the discordant group (149 IU/L [IQR, 128-224 IU/L]; *P* < .0001.

**Figure 2:**
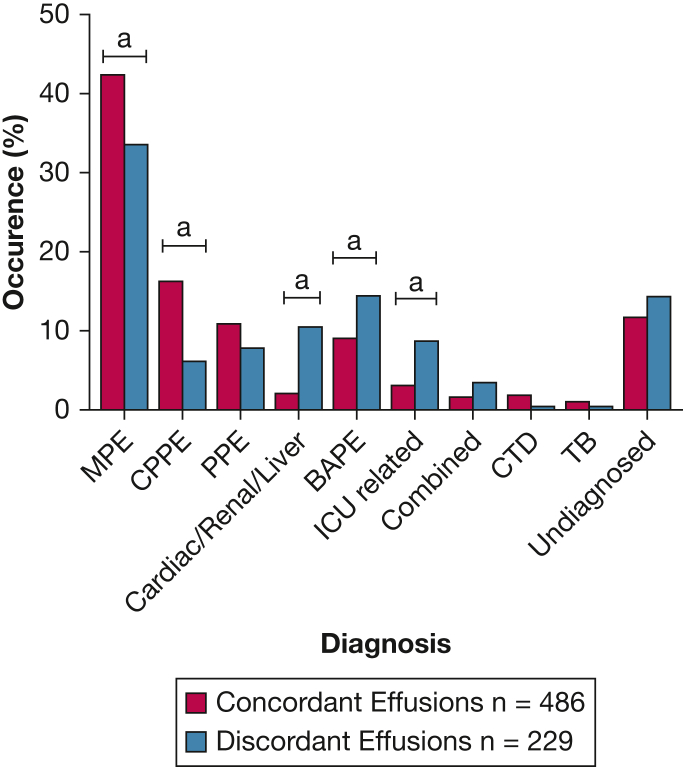
Frequency of each diagnosis in concordant vs discordant pleural effusion groups. ^a^Statistically significant difference between concordant and discordant groups. BAPE = benign asbestos-related pleural effusion; CPPE = complex parapneumonic pleural effusion or empyema; CTD = connective tissue disease; MPE = malignant pleural effusion; PF = pleural fluid; PPE = parapneumonic pleural effusion.

Table 1 and Figure 2 illustrate the frequency of each diagnosis and cancer subtype. Boxplots showing the distribution of age, pleural fluid protein, and pleural fluid LDH in each group are presented in e-Figure 1.

**Table 1:** Demographic Characteristics and Diagnostic Results of Patients With Concordant and Discordant Pleural Effusions

Characteristic	Concordant Effusions	Discordant Effusions
Age, y	70 (58-78)	75 (61-83)
Sex		
Female	193 (40)	118 (52)
Male	293 (60)	111 (48)
Diagnosis		
MPE	206 (42)	77 (34)
Primary tumor (% of all MPE)		
Lung	66 (32)	14 (18)
Breast	27 (13)	19 (25)
Mesothelioma	36 (17)	11 (14)
GI origin	21 (10)	10 (13)
Hematologic	9 (4)	9 (12)
Ovarian	18 (9)	4 (5)
Sarcoma	4 (2)	4 (5)
Other	25 (12)	6 (8)
CPPE or empyema	79 (16)	14 (6)
PPE	53 (11)	18 (8)
Fluid overload	10 (2)	24 (10)
Cardiac	8	23
Renal/hepatic	2	1
BAPE	44 (9)	33 (14)
ICU associated	15 (3)	20 (9)
Combined MPE + fluid overload	8 (2)	8 (3)
CTD	9 (2)	1 (0.5)
TB	5 (1)	1 (0.5)
Other/undiagnosed/insufficient data	57 (12)	33 (14)
Biochemistry		
PF protein, g/L	43 (37-47)	35 (27-42)
PF LDH, IU/L	494 (283-908)	149 (128-224)

Data are presented as median (interquartile range) or No. (%) unless otherwise indicated. BAPE = benign asbestos-related pleural effusion; CPPE = complex parapneumonic pleural effusion; CTD = connective tissue disease; GI = gastrointestinal; LDH = lactate dehydrogenase; MPE = malignant pleural effusion; PF = pleural fluid; PPE = parapneumonic pleural effusion.

### Diagnoses: All Discordant Pleural Effusions

Table 2 illustrates the relative frequency of each diagnosis in the concordant and discordant groups. The following results were observed when comparing concordant pleural effusions vs all discordant pleural effusions.

**Table 2:** Frequency of Pooled Diagnoses in the Concordant vs Discordant Groups

Diagnosis	In Concordant Effusions (n = 486)		In Discordant Effusions (n = 229)		*P* Value	OR (95% CI)	Adjusted *P* Value	Adjusted OR (95% CI)
MPE	206	42%	77	34%	.025^a^	0.69 (0.49-0.96)	.001^a^	0.57 (0.41-0.80)
Pleural infection (CPPE or empyema)	79	16%	14	6%	< .0001^a^	0.34 (0.29-0.61)	.002^a^	0.39 (0.21-0.71)
PPE	53	11%	18	8%	.20	0.70 (0.40-1.21)	.128	0.64 (0.36-1.13)
Fluid overload	10	2%	24	10%	< .0001^a^	5.57 (2.67-11.37)	< .0001^a^	5.37 (2.55-11.30)
Cardiac	8		23					
Renal/hepatic	2		1					
BAPE	44	9%	33	14%	.031^a^	1.69 (1.06-2.72)	.015^a^	1.88 (1.13-3.13)
ICU associated	15	3%	20	9%	.001^a^	3.01 (1.53-5.85)	.001^a^	3.32 (1.62-6.80)
Combined MPE + fluid overload	8	2%	8	3%	.81	0.89 (0.348-2.174)	.11	2.26 (0.82-6.25)
CTD	9	2%	1	0.5%	.13	0.23 (0.021 to 1.41)	.141	0.21 (0.03-1.68)
TB	5	1%	1	0.5%	.48	0.42 (0.036 to 3.07)	.603	0.53 (0.05-5.94)
Other/undiagnosed/insufficient data	57	12%	33	14%	.27	1.27 (0.79-2.00)	.237	1.34 (0.83-2.16)

*P* values were determined by using the Pearson χ^2^ test.

BAPE = benign asbestos-related pleural effusion; CPPE = complex parapneumonic pleural effusion; CTD = connective tissue disease; MPE = malignant pleural effusion; PPE = parapneumonic pleural effusion.

a Indicates significant *P* value. OR indicates diagnosis in discordant vs concordant group. Adjusted *P* values and ORs indicate *P* values and ORs when adjusted for age and sex in multivariable logistic regression analysis.

#### Fluid Overloaded States

Pleural effusions secondary to fluid overloaded states were defined as those secondary to cardiac, renal, and liver conditions, traditionally expected to cause a transudative pleural effusion. The proportion of pleural effusions due to fluid overloaded states was significantly greater in the discordant group: 24 (10%) of 229 compared with 10 (2%) of 486 in the concordant group (*P* < .0001, χ^2^ = 24.4, 1 *df*). The OR of a diagnosis relating to fluid overload with a discordant effusion compared with a concordant effusion was 5.57 (95% CI, 2.67-11.37). Following adjustments for age and sex, the association remained significant with an adjusted OR of 5.37 (95% CI, 2.55-11.30; *P* < .0001).

#### Malignancy

MPEs were defined as those in which malignancy was the primary cause of pleural effusion following analysis of biochemistry, cytology, imaging, and further tests such as histopathology. The proportion of malignant effusions was significantly lower in the discordant group (77 of 229 [34%] vs 206 of 486 [42%] in the concordant group; *P* = .025, χ^2^ = 5.0, 1 *df*). The OR for a malignant cause with a discordant effusion was 0.69 (95% CI, 0.49-0.96) compared with a concordant effusion. This association remained significant after adjusting for age and sex, with an adjusted OR of 0.57 (95% CI, 0.41-0.80; *P* = .001).

#### Pleural Effusions Related to Infective Diagnoses

Pleural effusions secondary to pleural infection were defined as complex parapneumonic pleural effusion (CPPE) and empyema. The frequency of pleural effusions due to pleural infection was significantly lower in the discordant group: 14 [6%] of 229 vs 79 [16%] of 486 (*P* < .0001, χ^2^ = 14.1, 1 *df*). The unadjusted OR for pleural infection was 0.34 (95% CI, 0.29-0.61) and remained significant after adjustment (adjusted OR, 0.39; 95% CI, 0.21-0.71; *P* = .002).

Simple parapneumonic pleural effusions (PPEs) were evaluated separately due to their differing biological and clinical characteristics. Simple PPE was present in 18 (8%) of 229 discordant effusions and in 53 (11%) of 486 concordant effusions, with no significant difference observed (OR, 0.70 [95% CI, 0.40-1.21 *P* = .20]; adjusted OR, 0.64 [95% CI, 0.36-1.13; *P* = .128]).

#### Other Nonmalignant Diagnoses

The incidence of BAPE was significantly higher in the discordant group (33 of 229 [14%] vs 44 of 486 [9%] in the concordant group; *P* = .031, χ^2^ = 4.7, 1 *df*). The unadjusted OR for BAPE was 1.69 (95% CI, 1.06-2.72) in the discordant group compared with an adjusted OR of 1.88 (95% CI, 1.13-3.13; *P* = .015) in the concordant group.

ICU-associated pleural effusions were classified based on intubated patients, with lack of clear ipsilateral parapneumonic pathology and requiring only a single drainage (no recurrence). These were higher in the discordant group, with 20 (9%) of 229 in the discordant group vs 15 (3%) of 486 in the concordant group (*P* = 0.001, χ^2^ = 10.7, 1 *df*). The OR of ICU-associated pleural effusion with a discordant effusion compared with a concordant effusion was 3.01 (95% CI, 1.53-5.85; adjusted OR, 3.32 [95% CI, 1.62-6.80; *P* = .001]).

No significant differences were observed in the rates of TB or connective tissue disease-related pleural effusion between the discordant and concordant groups (Table 1) prior to or following adjustment.

### Sensitivity Analysis

Sensitivity analysis was performed to account for missing pleural fluid protein and LDH values that precluded the classification into concordant or discordant effusion. When all missing pleural fluid biochemistry data were imputed either as “all missing values = concordant” or “all missing values = discordant,” the direction of ORs was maintained across all diagnoses. The statistically significant difference between concordant and discordant effusions in malignancy, fluid overload, pleural infection, and ICU-associated effusions remained significant regardless of whether missing values were treated as concordant or discordant. The significant association between discordance and BAPE was attenuated when all missing values were treated as concordant. The full results of the sensitivity analysis are included in e-Table 1.

### Subgroup Analysis

Further analysis was undertaken to elucidate trends between the subgroups of discordant pleural effusions, with assessment of protein-high discordant pleural effusions (with high protein, low LDH) and LDH-high discordant (with high LDH, low protein) separately. *P* values and ORs are described between discordant subgroup vs all concordant effusions. Subgroup analysis is further described in Table 3.

**Table 3:** Frequency of Diagnoses in Protein-High Discordant (LDH Low, Protein High) Pleural Effusions and LDH-High Discordant (Protein Low, LDH High) Effusions, With *P* Values vs Concordant Pleural Effusions

Diagnosis	In LDH-High Discordant Effusions (n = 85)		*P* Value (vs Concordant)	OR (95% CI)	In Protein-High Discordant Effusions (n = 144)		*P* Value (vs Concordant)	OR (95% CI)
MPE	18	21%	.0002^a^	0.37 (0.21-0.62)	59	41%	.76	0.94 (0.64-1.39)
Pleural infection (CPPE or empyema)	12	14%	.62	0.85 (0.44-1.65)	2	1%	< .0001^a^	0.073 (0.017-0.26)
PPE	8	9%	.68	0.85 (0.40-1.83)	10	7%	.16	0.61 (0.30-1.22)
Fluid overload	6	7%	.0099^a^	3.62 (1.28-9.58)	18	13%	< .0001^a^	6.80 (3.11-14.49)
Cardiac	5				18			
Renal/hepatic	1				0			
BAPE	10	12%	.56	0.75 (0.37-1.52)	23	16%	< .0001^a^	2.99 (1.90-4.96)
ICU associated	15	18%	< .0001^a^	6.73 (3.07-14.65)	5	3%	.81	1.29 (0.44-3.19)
Combined MPE + fluid overload	3	4%	.40	1.94 (0.55-7.23)	5	3%	.33	1.90 (0.70-5.50)
CTD	0	0%	.20	—	1	1%	.33	0.37 (0.034-2.26)
TB	0	0%	.37	—	1	1%	.33	0.75 (0.061-4.56)
Other/undiagnosed/insufficient data	13	15%	.27	1.44 (0.74-2.73)	20	14%	.36	1.29 (0.76-2.22)

*P* values were calculated by using the Pearson χ^2^ test. OR indicates diagnosis in the discordant subgroup vs the concordant group. Dashes (—) indicate OR not calculated due to frequency of 0 for 1 value. BAPE = benign asbestos-related pleural effusion; CPPE = complex parapneumonic pleural effusion; CTD = connective tissue disease; LDH = lactate dehydrogenase; MPE = malignant pleural effusion; PPE = parapneumonic pleural effusion.

a Indicates statistically significant result.

In diagnoses related to global fluid overload, the differences between protein-high and LDH-high subgroups vs concordant effusions were consistent, with 6 (7%) of 85 LDH-high discordant effusions due to fluid overload (OR, 3.62 [95% CI, 1.28-9.58; *P* = .0099] vs concordant effusions) and 18 (13%) of 144 protein-high discordant effusions due to fluid overload (OR, 6.80; 95% CI, 3.11-14.49; *P* < .0001).

MPEs were significantly less common in the LDH-high discordant group compared with concordant effusions: 18 (21%) of 85 (*P* = .0002; OR, 2.74; 95% CI, 1.61-4.74). The frequency of MPEs in the protein-high discordant group was not significantly different from the concordant group: 59 (41%) of 144 (*P* = .76).

Pleural infection remained significantly less frequent in protein-high discordant effusions compared with concordant effusions, with 2 (1%) of 144 (*P* < .0001) occurring due to pleural infection. Significant difference was not observed between LDH-high discordant effusions vs concordant effusions (12 [14%] of 85; *P* = .62).

No significant difference was found in the frequency of PPE compared with concordant effusions in either the LDH-high discordant group (8 of 85 [9%]; *P* = .68) or in the protein-high group (10 of 144 [7%]; *P* = .16). The incidence of BAPE was significantly lower in the protein-high group compared with the concordant group (23 of 144 [16%]; *P* < .0001). This effect was not observed in the LDH-high group compared with the concordant group (10 of 85 [12%]; *P* = .56). Analysis of all other subgroups revealed results consistent with the prior analysis between all discordant pleural effusions vs concordant, and the results are displayed in Table 3.

## Discussion

To our knowledge, this study is the largest to assess in detail the issue of discordant pleural effusions, with in-depth assessment of underlying demographic characteristics and diagnoses resulting in discordant pleural fluid biochemistry. In addition, the aim of this study was not to assess the performance of Light’s criteria, or the rate of “misclassification” of exudates, which other studies have sought to achieve,^8^ but to establish: (1) the true incidence of discordance in a large, real-world pleural practice; and (2) whether discordant pleural effusions represent a biologically discrete entity with distinct diagnostic patterns compared with concordant pleural exudates. Subanalysis on protein-high and LDH-high discordant effusions was undertaken to provide further mechanistic insights as to the diagnostic patterns observed.

This study found that discordance between LDH and protein in exudative pleural effusions is common, with up to 32% of exudative effusions displaying discordance. This is consistent with previously published smaller case studies.^11^ With the expansion in specialist pleural services, clinicians can be expected to encounter the clinical problem of discordant pleural fluid biochemistry, and establishing the associated diagnoses is of great importance.

We have shown that discordant exudates represent a cohort that behaves differently from concordant exudates, around which the majority of the current literature focuses and traditional diagnostic assumptions are based.^5^^,^^12^ This finding was robust in multivariable analysis and sensitivity analysis. In traditional concordant exudates, both LDH and protein are elevated, despite different underlying biological mechanisms by which each are released into pleural fluid. Pleural fluid protein is considered a biomarker of vascular permeability, whereas LDH typically denotes cellular turnover and inflammation.^13^, ^14^, ^15^ Diagnoses that cause both membranous infiltration in addition to high inflammatory load (eg, malignant pleural disease) can reasonably be expected to result in both high LDH and high protein in the pleural space. As such, typically “exudative” pleural effusions are those that result from either inflammatory or infiltrative processes, whereas transudative effusions are thought to result from increased hydrostatic pressure.^12^^,^^13^

In contrast, the biochemical mechanisms leading to discordance are unclear, although the results from this study may provide valuable insights. The increasing incidence of discordance with increased age may reflect an increase in vascular permeability with age.^16^

The current study results show that discordant effusions differ significantly from concordant pleural effusions in their underlying causes. The incidence of discordant pleural effusions resulting from global fluid overloaded states was significantly higher than in concordant effusions, a result that was consistent when analyzing subgroups. This finding could be due to a number of factors, including fluid concentration with diuretic use,^17^ or other causes of increased vascular permeability such as coexistent inflammation. However, the authors would suggest that in discordant effusions, underlying causes of fluid overload are actively investigated early in the pleural diagnostic pathway given these results.

The incidence of MPE was significantly lower in the discordant group, a result driven primarily by LDH-high discordant exudates. Protein-high discordant exudates did not differ significantly from concordant effusions in terms of MPE incidence, a result likely representing the cellular turnover and inflammation present in malignancy.

Discordant pleural effusions caused by pleural infection were significantly less common than in concordant pleural effusion, and this result was due in most part to the cohort with high protein, low LDH. As such, discordant exudates with low LDH are extremely rare, a finding biologically consistent with the underlying pathophysiology of infective effusions, which are typically among the most inflammatory pleural processes.^18^^,^^19^ In contrast, the frequency of simple PPEs was not significantly different from that of concordant effusions, illustrating the distinction between true pleural infection and simple PPEs. This is further reflected in their management, with PPE likely to resolve with antibiotic therapy alone.^20^

The incidence of BAPE was more frequent in the discordant group, specifically with high protein and low LDH. It is possible that this reflects the variable inflammation seen in this condition, which typically displays inflammation and/or fibrosis on histopathology.^21^^,^^22^ The increased frequency displaying low LDH may be reflective of a more fibrotic, less inflammatory process in these cases.

The other key diagnoses assessed revealed low numbers, precluding meaningful comparisons between groups, including tuberculous pleural effusions. This is a noteworthy point when extrapolating the data, as our center is situated in an area with low TB prevalence.

With the increasing incidence of pleural disease, and evidence revealing the high cost associated with pleural effusion management,^23^ the authors suggest that this study emphasizes the importance of identifying discordant exudates early in the pleural diagnostic pathway to guide onward investigations and to minimize delays in management of the underlying condition.

The current study has several key limitations that must be considered in wider extrapolation. Because the study used a retrospective cohort, the rate of dual diagnoses may have been underestimated, although the frequencies seemed well matched between concordant and discordant groups. Similarly, the concurrent use of diuretics in patients with a diagnosis of fluid overload, while similar between transudates and discordant exudates, is challenging to quantify retrospectively, and corrections for subtle variations in dosing may be difficult. The single-center nature of the study may limit wider application, in particular to centers with higher rates of tuberculous or infective pleural effusions, and as such the results are more applicable to a Western/European context. The authors additionally acknowledge that careful history and examination, in conjunction with other tests (eg, pleural biopsy), may provide diagnostic information early in the pathway, reducing the usefulness in these cases of observing discordance. Finally, serum protein and LDH were sent infrequently in this cohort and, as such, this study is unable to assess the impact of serum parameters on discordance. Future multicenter prospective study to assess discordance would provide further validation of these findings.

## Interpretation

The data from this large, deeply phenotyped population show that discordant pleural exudates are common, occurring in slightly less than one-third of all exudative pleural effusions. Discordant pleural effusions appear to have a distinct diagnostic pattern, which clinicians should be aware of when investigating and managing patients with discordant pleural fluid biochemistry. Future studies should assess how discordance or concordance correlates with ability to reclassify an exudate as a transudate compared with other measurements currently in use such as albumin and cholesterol.

## Funding/Support

The authors have reported to *CHEST* that no funding was received for this study.

## Financial/Nonfinancial Disclosures

None declared.

## Supplementary Data

e-Online Data

e-Online Data

e-Online Data
