# Supplementary figures and images for: Incidence of Discordant Pleural Fluid Exudates and Diagnostic Patterns: A Retrospective Cohort Study
Source: Chest. 2025 Jun 28;168(6):1517–27. doi: 10.1016/j.chest.2025.05.048 (PMC12833483; doi:10.1016/j.chest.2025.05.048)

e-Figure 1:

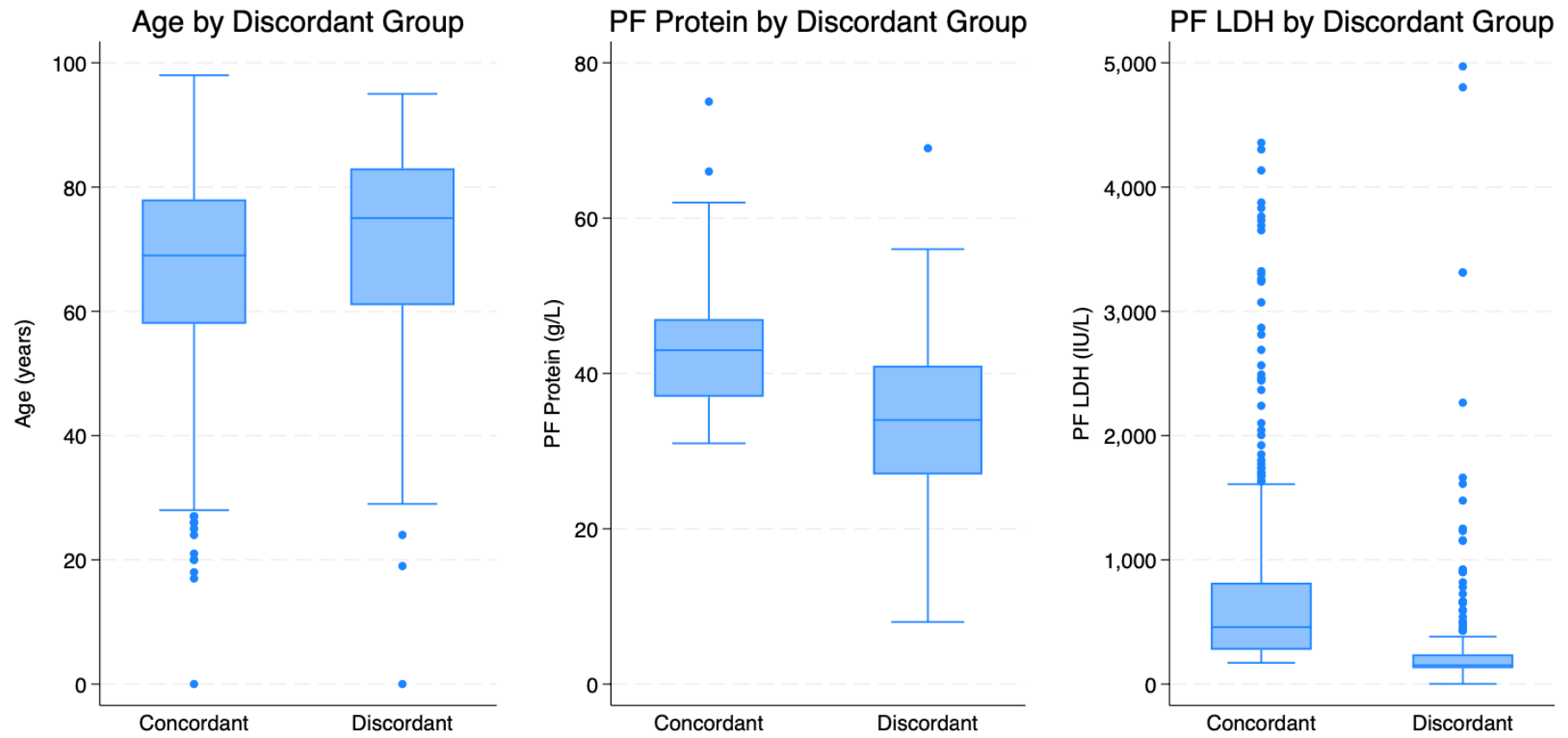

Supplement: e-Online Data [file mmc1.pdf]
