# Supplementary material for: Incidence of Discordant Pleural Fluid Exudates and Diagnostic Patterns: A Retrospective Cohort Study
Source: Chest. 2025 Jun 28;168(6):1517–27. doi: 10.1016/j.chest.2025.05.048 (PMC12833483; doi:10.1016/j.chest.2025.05.048)
Supplement: e-Online Data [file mmc2.docx]

**e-Table 1**

| Diagnosis | Complete case analysis, n=715 | | | Sensitivity analysis: missing values recorded as 'discordant', n= 804 | | | Sensitivity analysis: missing values recorded as 'concordant', n=804 | | |
| --- | --- | --- | --- | --- | --- | --- | --- | --- | --- |
|  | Adjusted Odds ratio | 95% CI | P value | Adjusted odds ratio | 95% CI | P value | Adjusted odds ratio | 95% CI | P value |
| MPE | 0.57 | 0.41–0.80 | 0.001 | 0.51 | 0.38–0.70 | <0.0001 | 0.66 | 0.47–0.92 | 0.015 |
| CPPE | 0.39 | 0.21–0.71 | 0.002 | 0.40 | 0.24–0.68 | 0.001 | 0.43 | 0.23–0.78 | 0.006 |
| PPE | 0.64 | 0.36–1.13 | 0.128 | 0.57 | 0.34–0.97 | 0.035 | 0.73 | 0.41–1.27 | 0.266 |
| Fluid Overload | 5.37 | 2.55–11.30 | <0.0001 | 5.77 | 2.89–11.54 | <0.0001 | 2.48 | 1.38–4.47 | 0.002 |
| BAPE | 1.88 | 1.13–3.13 | 0.015 | 1.32 | 0.81–2.15 | 0.260 | 2.12 | 1.29–3.51 | 0.003 |
| ICU associated | 3.32 | 1.62–6.80 | 0.001 | 3.59 | 1.86–6.93 | 0.001 | 2.33 | 1.24–4.39 | 0.009 |
| Combined MPE + Fluid Overload | 2.26 | 0.82–6.25 | 0.114 | 1.73 | 0.65–4.58 | 0.273 | 2.27 | 0.85–6.07 | 0.101 |
| CTD | 0.21 | 0.03–1.68 | 0.141 | 0.31 | 0.07–1.45 | 0.136 | 0.23 | 0.03–1.81 | 0.162 |
| TB | 0.53 | 0.05–5.94 | 0.603 | 0.91 | 0.14–5.90 | 0.920 | 0.58 | 0.05–6.22 | 0.649 |
| Other/Undiagnosed | 1.34 | 0.83–2.16 | 0.237 | 1.90 | 1.26–2.87 | 0.002 | 1.04 | 0.66–1.63 | 0.871 |

e-Table 1: Sensitivity analysis for missing pleural fluid protein or LDH values (n=89). Adjusted odds ratio, 95% confidence intervals and p-values shown for complete case analysis, ‘conservative’ estimates of missing data (all missing = concordant) and ‘liberal’ estimates (all missing = discordant). All odds ratios adjusted for age and sex.

Abbreviations: MPE, malignant pleural effusion; CPPE, complex parapneumonic pleural effusion; PPE, simple parapneumonic pleural effusion; BAPE, benign asbestos related pleural effusion; CTD, connective tissue disease; TB, tuberculosis.
